# Associations between Cognitive, Sociocontextual, and Affective Variables and Unprotected Anal Intercourse among Men Who Have Sex with Men—A Comparative Study Conducted in Two Chinese Cities

**DOI:** 10.1155/2014/970975

**Published:** 2014-03-05

**Authors:** Joseph T. F. Lau, T. J. Feng, X. L. Liu, Jing Gu, Hi Yi Tsui, F. C. Hong, Zixin Wang, Wangnan Cao

**Affiliations:** ^1^Centre for Health Behaviors Research, School of Public Health and Primary Care, Faculty of Medicine, The Chinese University of Hong Kong, Hong Kong; ^2^Centre for Medical Anthropology and Behavioral Health, Sun Yat-sen University, Guangzhou 510080, China; ^3^CUHK Shenzhen Research Institute, Shenzhen 518057, China; ^4^School of Public Health and Primary Care, Prince of Wales Hospital, Shatin, Hong Kong; ^5^Shenzhen Center for Chronic Disease Control, Shenzhen 518020, China; ^6^School of Public Health, Sun Yat-sen University, Guangzhou 510080, China

## Abstract

Few studies compared HIV-related risk behaviors between cities with different sociocultural environments among men who have sex with men (MSM). This study investigated the prevalence of unprotected anal intercourse (UAI) and associated individual and socio-cultural factors among Chinese MSM in Hong Kong and Shenzhen in Mainland China, which were proximal to each other but experienced different socioeconomic developments. Amongst all the 535 participants, 40.2% had had UAI. Significant factors of UAI among Shenzhen MSM included being able to find someone to share one's sexual orientation, disclosure of sexual orientation to family members, HIV risk perception, and use of alcohol or substances (adjusted OR ranged from 2.37 to 4.91), whilst disclosure of sexual orientation to family members was the only significant factor among Hong Kong MSM (adjusted OR = 1.64). Geographic variations in factors associated with UAI were observed. Future research and interventions need to take this into account.

## 1. Introduction

HIV prevalence among men who have sex with men (MSM) has been increasing sharply in China [1]. A number of Chinese cities (e.g., Chongqing and Chengdu) reported HIV prevalence among MSM exceeding 10% [2, 3] and HIV incidence higher than 5 per 100 person-years [4]. Moreover, risk behaviors associated with HIV infection such as unprotected anal intercourse (UAI) with different types of sex partners and multiple sex partnership [2, 5–10] are prevalent among MSM in China. Prevalent bisexual behaviors draw the attention that MSM spread HIV to the general female population [7–9].

Risk factors associated with UAI among MSM in China are multidimensional. Such factors include cognitive factors (e.g., HIV-related knowledge, risk perceptions, and perceived condom efficacy in preventing HIV) [10–14], contextual factors (e.g., condom availability and use of alcohol or psychoactive substances) [15, 16], sociocultural factors such as social support and stigma [14, 17], and affective factors such as internalized homophobia [18]. Traditional Chinese culture does not find MSM behaviors acceptable [19]. Many MSM in China had encountered stigma [2, 19]. Effective interventions need to take all these multidimensional factors into account.

This study compared the prevalence of UAI and associated factors among MSM in two cities of China: Hong Kong and Shenzhen. Hong Kong is contiguous to Shenzhen, with travel time of about one hour. Millions of travelers commute daily between the two cities. About 15% of the Hong Kong MSM travel to Shenzhen to seek male sex partners [20]. There is no similar data on how many Shenzhen MSM travel to Hong Kong for the same purpose. Nevertheless, we expect the number to be a large one as it is convenient to do so and Hong Kong is a very popular destination for mainlanders to visit. The two cities have some common grounds to facilitate comparisons. First, the HIV prevalence among MSM is comparable as it was 4.06% in Hong Kong [21] and 2.6% in Shenzhen in 2006 [22]. Second, homosexuality is currently not criminalized in both cities. In contrast, Hong Kong has a longer history of exposure to international interactions. Although discrimination against MSM is still prevalent in Hong Kong [23], the social environment surrounding MSM seems to be more open than that of Shenzhen and the gay community in Hong Kong seems to be better developed than that in Shenzhen. For instance, laws against discrimination based on sexual orientation [24], advocacy groups for gay rights, and public annual activities such as gay movie festivals exist in Hong Kong but not in Shenzhen. In Shenzhen, the only apparent gay community is that of a nongovernmental organization working on HIV prevention.

This study investigated the prevalence of UAI among MSM in Shenzhen and in Hong Kong. Associations between UAI and socioecological factors were identified separately for the Shenzhen and the Hong Kong samples. The results of the present study allow us to understand better risk factors of UAI and facilitate design of effective interventions. Some of such interventions may need to tackle prevalent cross-border risk behaviors [25] and hence may need to target MSM both in Hong Kong and in Shenzhen. The study also contributes to our understanding of degree of homogeneity of risk factors in various cities within a country that have both similarities and differences, reminding researchers of cautions in making generalization to big countries such as China from results that are obtained from a single city. These comparisons provide some insights into how the social environment affects risk behaviors among MSM.

## 2. Methods

### 2.1. Study Populations and Sampling

All respondents were Chinese males of age ≥18 years old, who self-reported having had anal intercourse with at least one man in the last 12 months and had never been engaged in sex work. Snowball sampling methods were used in the surveys. Respondents were recruited from local gay venues (four gay bars and five saunas in Shenzhen; six gay bars and five saunas in Hong Kong) and gay websites (only for the Hong Kong sample). Prospective respondents were briefed about the details of the study. With informed consent, face-to-face interviews were administered by experienced and well-trained interviewers, who were staff of the Shenzhen Chronic Disease Hospital (Shenzhen sample) or peer fieldworkers in Hong Kong. The questionnaire was written in Chinese, which is the same for the two samples. The interviews were administered in Mandarin in Shenzhen and Cantonese in Hong Kong, as those are spoken by the two samples, respectively; the contents are however the same and comparability was ensured. Respondents were given about US$6.4 as a compensation for their time spent in the study. Ethics approval was obtained from the Ethics Committee of the Chinese University of Hong Kong. The response rate was about 70%.

### 2.2. Measures

The dependent variable of this study was whether having had at least one episode of UAI with the same sex in the last 12 months. There are five blocks of potential factors: (1) background variables including sociodemographics, data on sexual orientation and sexual behaviors, and self-reported STD infection in the last 12 months; (2) cognitive variables including three variables that were related to HIV-related knowledge, perceived chance of contracting HIV in the future (risk perception), perceived efficacy of condom use in preventing HIV transmissions, and perceived discrimination toward MSM; (3) contextual factors including always drink alcohol before having sex and substance use in the last 12 months; (4) social factors including being able to find someone to share feelings about one's sexual orientation and disclosure of his sexual orientation to family members; and (5) affective factors including afraid of others knowing his MSM status, acceptance of his sexual orientation, and perceived negative feelings associated with MSM status (whether the respondent associated his MSM status with worry, shame, social exclusion, family/peer pressure, and fear of contracting HIV). Substance use was assessed by asking whether the participant had used psychiatric substances. A question asked about local residency is defined as having the right of abode in Hong Kong (Hong Kong respondents) and holding a registered residence card (Hukou) for Shenzhen respondents.

### 2.3. Statistical Analyses

The distributions of the independent variables and the dependent variable were compared by using univariate odds ratios (OR) and 95% confidence intervals (CI). Those sociodemographic variables that were univariately significantly associated with the dependent variable were entered into a multivariate stepwise logistic regression model; significant variables were adjusted for in subsequent analyses. Adjusted OR and respective 95% CI were reported. Multiplicative interactions between the independent variables and the location variable were tested by using multiple logistic regression analyses. SPSS 15.0 was used for data analyses and *P* value < 0.05 was taken as statistically significant.

## 3. Results 

### 3.1. Between-City Differences in the Distributions of the Independent Variables

As compared to Hong Kong respondents, Shenzhen respondents were more likely to belong to the age group “25–34,” to have a lower education level, to be ever married, to be currently unemployed or not being a student, and to be nonlocals (*P* < 0.01). Shenzhen respondents were also more likely than Hong Kong respondents to have had bisexual behaviors, self-identified as a bisexual person, and self-reported STD infection in the last 12 months (*P* < 0.01; Table 1).

Some conditions seemed to favor Shenzhen respondents, who were more likely than their Hong Kong counterparts to know about the asymptomatic property of HIV transmission (% giving incorrect responses—Shenzhen: 13.7% versus Hong Kong: 21.7%, adjusted OR = 0.36, *P* < 0.01), less likely to perceive discrimination toward MSM (Shenzhen: 31.7% versus Hong Kong: 61.0%, adjusted OR = 0.37, *P* < 0.01), and less likely to disclose their sexual orientation to none or only some (but not all) family members (Shenzhen: 28.4% versus Hong Kong: 48.5%, adjusted OR = 0.39, *P* < 0.01; Table 2).

Other significant differences between the two cities did not favor Shenzhen respondents, who showed higher likelihood of (1) giving incorrect responses to two of the three HIV-related knowledge questions (adjusted OR = 2.53 and 5.02, resp., *P* < 0.01), (2) afraid of others knowing their MSM status (Shenzhen: 78.4% versus Hong Kong: 61.9%, adjusted OR = 2.85, *P* < 0.01), and (3) giving ≥3 affirmative responses to the five perceived negative feelings (worry, shame, social exclusion, family/peer pressure, and afraid of contracting HIV) associated with one's MSM status (Shenzhen: 68.6% versus Hong Kong: 48.4%, adjusted OR = 3.02, *P* < 0.01) whilst lower likelihood of (1) being able to find someone to share feelings about their sexual orientation (Shenzhen: 30.7% versus Hong Kong: 57.6%, adjusted OR = 0.32, *P* < 0.01) and (2) accepting their sexual orientation (Shenzhen: 67.6% versus Hong Kong: 90.0%, adjusted OR = 0.19, *P* < 0.01). The results of the univariate analysis are similar to those of the adjusted analysis and are presented in related tables.

### 3.2. Factors Associated with UAI among Shenzhen and Hong Kong Respondents

Education level and age group remained significant in the multivariate analysis among the Shenzhen and the Hong Kong respondents (data not tabulated). These two variables were adjusted for when investigating the respective associations between different factors and UAI in the two groups.

Adjusted for the significant background variables, disclosure of one's sexual orientation to family members was the only variable that was significantly associated with UAI in both samples. The association was stronger for Shenzhen respondents as compared to Hong Kong respondents (adjusted OR = 3.66 and 1.64, resp.; *P* value for the interaction between this variable and the location variable was 0.043; data not tabulated). In similar adjusted analysis, some independent variables were significantly associated with UAI in the Shenzhen sample but not in the Hong Kong sample. Such variables included (1) perceived chance of contracting HIV in the future, (2) always drinking alcohol before having sex, (3) using psychoactive substances, and (4) able to find someone to share feelings about his sexual orientation (Table 3). The interaction terms between the location variable and the aforementioned independent variables, adjusted for relevant sociodemographic variables, were all significant (*P* < 0.05; data not tabulated). No independent variable was significant in the Hong Kong sample but not in the Shenzhen sample in the adjusted analysis. The other factors were nonsignificant in both samples in the adjusted analysis (*P* > 0.05; see Table 3).

## 4. Discussion

There are both commonality and differences when the two samples were compared. In terms of similarities, the two cities showed some similar epidemiological data such as prevalence of high risk behaviors (UAI, large number of sex partners, and use of substances and alcohol before having sex with men) and risk perception. Without a deeper look, programmers may erroneously come to the conclusion that similar HIV prevention strategies can be applied to these two cities. The sociocultural contexts are however different in some aspects that may lead to disparities.

Regarding differences, we found that Shenzhen MSM were less likely than Hong Kong MSM to perceive discrimination toward MSM. This observation was made despite the impression that Hong Kong seems to be a more westernized and open society. It is possible that Shenzhen MSM had lower awareness of their rights. For instance, Hong Kong has an antidiscriminatory act to protect the rights of having different sexual orientation and the Equal Opportunities Commission, which is a statutory body to oversee implementation of the antidiscriminatory act. It is possible that an episode that is seen as discriminatory toward MSM among Hong Kong MSM is not seen to be a case among their Shenzhen counterparts. Higher media coverage on discrimination topics in Hong Kong may also account for the difference. The literature on whether perceived discrimination affects UAI is mixed [11, 13, 26] and the association was nonsignificant in our case.

Furthermore, our Shenzhen respondents were less knowledgeable about some HIV-related knowledge items as compared to the Hong Kong respondents; our results show that such knowledge was not associated with UAI. The two samples did not differ in prevalence of perceived efficacy for using condoms to prevent HIV transmission; the variable was also not significantly associated with UAI in both samples. In the literature, the results about associations between HIV-related knowledge or some cognitive factors and UAI have been mixed [27, 28]. In many parts of China, HIV prevention targeting MSM is information-based. Our results suggest that although such campaigns are easy to implement, their effectiveness is not well supported by evidence.

The prevalence of self-reported STD was higher among Shenzhen MSM than among Hong Kong MSM. Surveillance data showed that the prevalence of syphilis among Shenzhen MSM was as high as 20% [22]. There is hence a strong need to integrate HIV and STD prevention targeting MSM in Mainland China, and some reports claimed that the two systems are separated from each other. Self-reported STD history was not associated with UAI; the risk of recurrent STD infections is high. Counseling for STD patients is warranted.

Although the level of risk perception was comparable in the two samples, it was significantly associated with UAI among Shenzhen MSM, but not the Hong Kong sample. Risk perception (perceived susceptibility) is an important component of many health behavioral theories, such as the Health Belief Model [29], and was associated with UAI in some studies though the results were not conclusive [30]. The concept is the foundation of many HIV campaigns targeting MSM—informing them that they are at risk. Our results suggested that the approach would be more effective in Shenzhen than in Hong Kong. The results further bring attention to health behavioral theorists that it is not necessary that similar associations would be observed even in two societies with some similarities. It is known that theory-based HIV prevention programs are more likely to be effective as compared to nontheory-based ones [31]. However, caution should be given when using these models to guide intervention planning as geographic variations may be substantial. We cannot explain the differential associations and further studies are warranted.

Similarly, the prevalence of using substances or alcohol showed no statistically significant between-city differences but interaction effects were observed—significant associations with UAI were found in Shenzhen but not in Hong Kong. We do not know whether the differential is due to differences in the type and dosage of substances/alcohol used, settings, or ability to control oneself. Again, it cautioned us about generalization of risk factors obtained from a single city to a country, which often occurred in the literature.

Shenzhen MSM showed lower levels of acceptance and sharing, but higher level of fear for disclosure and affective distress associated with their same-sex sexual behaviors and sexual orientation. Although sexual orientation was not associated with UAI in both samples, variables related to sharing and nondisclosure related to sexual orientation were significantly associated with UAI in the Shenzhen but not in the Hong Kong sample. It suggests that it is not sexual orientation that affects risk behaviors, but it is feelings and discomfort attached to the sexual orientation that matter. In Hong Kong, there is more social space for MSM and many celebrities disclose their gay status in public. It may be easier for Hong Kong MSM to come out of the closet and form a clear identity that matches with their sexual orientation. Gay movements are very new in Mainland China. Many MSM in China migrated recently from smaller towns or rural areas to metropolitan areas [32]. To live with a MSM life-style in a new environment is an adaptation process and confusion might arise. Support groups about the coming out process and counseling services are warranted and should be considered as part of the intervention programs in China.

The study has a number of limitations. The sampling may not be representative and respondent-driven sampling may be a better design. Responses are subject to self-report bias due to social desirability. Response rate was 70%, which is comparable to other similar studies [27, 33, 34]; refusal may be related to time issue, research topic, or other reasons. Gay websites were used for recruitment in Hong Kong but not in Shenzhen as we were not able to contact keepers of websites in Shenzhen; some sampling bias may exist. The sampling excluded male sex workers as they may have different associated factors; such decision should have improved data interpretation but may also limit generalization. The fact that different people were used to interview participants at each site was another limitation, which may cause bias when comparing the differences between sites. The sample sizes of the two groups were different and hence the power for the logistic regression analysis for factors associated with UAI; the large differences in the measures of associations (i.e., adjusted odds ratios) and the significant interaction terms, however, suggest that the difference in power of the two samples would be unable to explain the large between-cities differences. Indicator questions, instead of validated scales, were used in this study, though many of these items have been used in previous studies [20, 27].

In sum, the surveys were comparable as they used the same question items. It shows that sociocontextual variables are potentially important in explaining UAI among MSM in Mainland China. The important findings included that the associations between such variables and UAI could vary dramatically between societies, even those which are relatively comparable such as Hong Kong and Shenzhen. It also calls for caution in interpretation of results and applications of behavioral theories. Generalization of research findings should therefore be made carefully. Identification with one's sexual orientation is potentially a huge problem among MSM in China and counseling services are warranted. More importantly, HIV intervention programs targeting MSM need to be evidence-based and would benefit from sound research support.

## Figures and Tables

**Table 1:** Comparison of background factors between Shenzhen and Hong Kong respondents.

	All (*n* = 535)	Shenzhen (*n* = 102)	Hong Kong (*n* = 433)	Shenzhen versus Hong Kong	
	Col%	Col%	Col%	ORu^1^ (95% CI)	ORadj^2^ (95% CI)
*Sociodemographics *					
Age group					
18–24	35.3	20.2	38.8	1.00	1.00
25–34	53.0	70.7	49.0	2.77 (1.62, 4.74)**	2.35 (1.25, 4.40)**
35–52	11.7	9.1	12.2	1.43 (0.61, 3.32)	0.58 (0.20, 1.70)
Education level					
Junior high and below	6.2	16.0	3.9	1.00	1.00
Senior high	55.2	71.0	51.5	0.34 (0.16, 0.70)**	0.66 (0.28, 1.55)
University and above	38.6	13.0	44.6	0.07 (0.03, 0.17)**	0.11 (0.04, 0.29)**
Marital status					
Never married	95.9	87.9	97.7	1.00	1.00
Ever married (e.g., divorced, widow)	4.1	12.1	2.3	5.83 (2.44, 13.93)**	7.72 (2.34, 25.48)**
Employment					
Full- or part-time	74.6	74.7	74.6	1.00	1.00
Not employed	9.2	23.2	6.0	3.86 (2.09, 7.14)**	3.21 (1.53, 6.75)**
Student	16.2	2.0	19.4	0.10 (0.02, 0.43)**	0.18 (0.04, 0.80)*
Local residence					
No	3.8	12.0	1.8	1.00	1.00
Yes	96.2	88.0	98.2	0.14 (0.05, 0.35)**	0.19 (0.07, 0.58)**

*Sexual orientation and sexual behaviors *					
UAI with men in the last 12 months					
No	59.8	59.8	59.8	1.00	1.00
Yes, UAI	40.2	40.2	40.2	1.00 (0.64, 1.55)	1.09 (0.64, 1.85)
Number of MSM partners in the last 12 months					
1–3	55.0	50.0	56.1	1.00	1.00
≥4	45.0	50.0	43.9	1.28 (0.83, 1.97)	1.24 (0.74, 2.07)
Had sex with females in the last 12 months					
No	92.5	74.5	96.8	1.00	1.00
Yes	7.5	25.5	3.2	10.24 (5.11, 20.50)**	5.46 (2.33, 12.81)**
Sexual orientation					
Homosexual	77.3	59.4	81.5	1.00	1.00
Bisexual	18.7	33.7	15.2	3.03 (1.85, 4.98)**	2.49 (1.33, 4.65)**
Not sure	3.9	6.9	3.2	2.94 (1.14, 7.59)*	2.40 (0.73, 7.88)

*STD infection *					
Self-reported STD (last 12 months)					
No	96.0	88.8	97.7	1.00	1.00
Yes	4.0	11.2	2.3	5.35 (2.20, 12.98)**	6.96 (2.35, 20.61)**

**P value < 0.01, *P value < 0.05.

^1^Odds ratio of univariate analysis.

^2^For the analysis of the set of sociodemographic variables, adjusted odds ratios were obtained by applying stepwise multivariate analysis using variables with *P* < 0.05 in the univariate analysis as candidates. Regarding the rest of the tables, adjusted odds ratios were obtained from multivariate analysis adjusted for those sociodemographic variables which were found to be significant in the stepwise multivariate analysis.

**Table 2:** Comparison of cognitive, contextual, social, and affective factors between Shenzhen and Hong Kong respondents.

	All (*n* = 535)	Shenzhen (*n* = 102)	Hong Kong (*n* = 433)	Shenzhen versus Hong Kong	
	Col%	Col%	Col%	ORu^1^ (95% CI)	ORadj^2^ (95% CI)
*Cognitive factors *					
HIV-related knowledge					
(i) Infectivity of a healthy-looking HIV-infected person					
Correct	79.8	86.3	78.3	1.00	1.00
Incorrect	20.2	13.7	21.7	0.57 (0.31, 1.05)^+^	0.36 (0.17, 0.76)**
(ii) Infectivity via kissing with a HIV-infected person					
Correct	59.1	44.1	62.6	1.00	1.00
Incorrect	40.9	55.9	37.4	2.12 (1.37, 3.28)**	2.53 (1.50, 4.27)**
(iii) Detection of HIV one week after infection took place					
Correct	74.4	44.1	81.5	1.00	1.00
Incorrect	25.6	55.9	18.5	5.59 (3.53, 8.85)**	5.02 (2.89, 8.70)**
Perceived chance of contracting HIV in the future					
Extremely low/low	65.2	66.3	64.9	1.00	1.00
Moderate/high/extremely high	34.8	33.7	35.1	0.94 (0.59, 1.48)	0.75 (0.44, 1.28)
Perceived efficacy of condom use for HIV prevention					
Extremely low/low/moderate	14.2	17.8	13.4	1.00	1.00
High/extremely high	85.8	82.2	86.6	0.71 (0.40, 1.27)	1.51 (0.74, 3.06)
Perceived discrimination toward MSM					
No/a little	44.6	68.3	39.0	1.00	1.00
Some/very much	55.4	31.7	61.0	0.30 (0.19, 0.47)**	0.37 (0.21, 0.64)**

*Contextual factors (last 12 months) *					
Always drink alcohol before having sex					
No	84.7	81.2	85.5	1.00	1.00
Yes	15.3	18.8	14.5	1.36 (0.76, 2.42)	0.72 (0.36, 1.45)
Use of psychotropic substances					
No	84.7	78.6	86.1	1.00	1.00
Yes	15.3	21.4	13.9	1.70 (0.97, 2.95)^+^	1.19 (0.63, 2.27)

*Social factors *					
Can find someone to share sexual orientation					
No	27.4	36.6	25.2	1.00	1.00
Yes	52.5	30.7	57.6	0.37 (0.22, 0.62)**	0.32 (0.17, 0.59)**
Do not feel such a need	20.1	32.7	17.1	1.31 (0.75, 2.29)	1.19 (0.60, 2.36)
Disclosure of sexual orientation to family members					
Yes, disclosed to all family members	55.3	71.6	51.5	1.00	1.00
Disclosed to none or only some family members	44.7	28.4	48.5	0.42 (0.26, 0.67)**	0.39 (0.23, 0.67)**

*Affective factors *					
Afraid of others knowing his MSM status					
No	35.0	21.6	38.1	1.00	1.00
Yes	65.0	78.4	61.9	2.24 (1.34, 3.73)**	2.85 (1.59, 5.11)**
Accept one's sexual orientation					
No/almost no	14.2	32.4	10.0	1.00	1.00
Yes	85.8	67.6	90.0	0.23 (0.14, 0.39)**	**0.19 (0.10, 0.37)∗∗**
Perceived negative feelings associated with MSM status^@^					
≤2 kinds of impacts	47.8	31.4	51.6	1.00	1.00
3~5 kinds of impacts	52.2	68.6	48.4	2.33 (1.44, 3.84)**	3.02 (1.76, 5.20)**

**P value < 0.01, *P value < 0.05, and ^+^ *P* value < 0.1.

^1^Odds ratio of univariate analysis.

^2^Odds ratio adjusting for sociodemographic variables significant in the stepwise multivariate analysis in Table 1.

^@^A variable was generated by combining responses to the 5 individual items related to perceived negative feelings associated with one's MSM status (worry, shame, social exclusion, family/peer pressure, and afraid of contracting HIV).

ORs and 95% CIs with *P* < 0.05 were in bold.

**Table 3:** Factors associated with UAI with men in the last 12 months among the Shenzhen and the Hong Kong respondents.

	UAI in the last 12 months					
	Shenzhen (*n* = 102)			Hong Kong (*n* = 433)		
	Row %	ORu^1^ (95% CI)	ORadj^2^ (95% CI)	Row %	ORu^1^ (95% CI)	ORadj^3^ (95% CI)
*Cognitive factors *						
Perceived chance of contracting HIV in the future						
Extremely low/low	28.4	1.00	1.00	41.3	1.00	1.00
Moderate/high/extremely high	64.7	**4.63 ** **(1.92**–11.18)**	3.91 (1.57, 9.70)**	38.2	0.88 (0.59, 1.32)	0.90 (0.60, 1.36)

*Contextual factors *						
Always drink alcohol before having sex						
No	32.1	1.00	1.00	39.2	1.00	1.00
Yes	72.2	5.51 (1.77, 17.16)**	4.91 (1.53, 15.75)**	46.0	1.32 (0.77, 2.27)	1.46 (0.84, 2.52)
Use of psychotropic substances						
No	32.5	1.00	1.00	39.7	1.00	1.00
Yes	66.7	4.16 (1.49, 11.59)**	3.23 (1.09, 9.57)*	43.3	1.16 (0.67, 2.02)	1.18 (0.67, 2.07)

*Social factors *						
Can find someone to share sexual orientation						
No	29.7	1.00	1.00	41.3	1.00	1.00
Yes	58.1	3.27 (1.20, 8.92)*	4.00 (1.36, 11.73)*	37.8	0.86 (0.54, 1.37)	0.92 (0.58, 1.48)
Do not feel such a need	33.3	1.18 (0.43, 3.25)	1.38 (0.48, 4.01)	45.9	1.21 (0.67, 2.19)	1.33 (0.72, 2.46)
Disclosure of sexual orientation to family members						
Yes, disclosed to all family members	31.5	1.00	1.00	35.0	1.00	1.00
Disclosed to none or only some family members	62.1	3.56 (1.45, 8.73)**	3.66 (1.41, 9.53)**	45.7	1.57 (1.06, 2.30)*	1.64 (1.10, 2.44)*

^1^Odds ratio of univariate analysis.

^2^Odds ratio adjusting for “education level” which is significant in the stepwise multivariate analysis (data not tabulated).

^3^Odds ratio adjusting for “age group” which is significant in the stepwise multivariate analysis (data not tabulated).

**P value < 0.01, *P value < 0.05, and ^+^ *P* value < 0.1.

Variables considered but not significant in either samples included (1) number of MSM partners, (2) bisexual behaviors in the last 12 months, (3) sexual orientation, (4) self-reported STD infection, (5)–(7) the three items on HIV-related knowledge (“Infectivity via kissing with a HIV-infected person” and “Detection of HIV one week after infection took place” were marginally significant in the Shenzhen sample), (8) perceived efficacy of condom use for HIV prevention, (9) perceived discrimination toward MSM, (10) afraid of others knowing his MSM status (marginally significant in the Shenzhen sample), (11) acceptance of one's sexual orientation (marginally significant in the Shenzhen sample), and (12) perceived negative feelings associated with MSM status (worry, shame, social exclusion, family/peer pressure, and afraid of contracting HIV) related to one's sexual orientation.
